# 2435. Stewardship – It’s Not Just Antibiotics and Testing

**DOI:** 10.1093/ofid/ofad500.2054

**Published:** 2023-11-27

**Authors:** Mindy Sampson, Catherine Passaretti, Siobhan Eichenblat, Shelley Kester, Jessica Layell, Laura Noonan, Corrianne Billings, Werner Bischoff

**Affiliations:** Wake Forest University School of Medicine, Charlotte, North Carolina; Advocate Health, Charlotte, NC; Atrium Health, Charlotte, North Carolina; Atrium Health, Charlotte, North Carolina; Atrium Health, Charlotte, North Carolina; Atrium Health, Charlotte, North Carolina; Atrium Health Wake Forest Baptist, Winston Salem, North Carolina; Wake Forest University School of Medicine, Winston Salem, NC

## Abstract

**Background:**

Longer central line (CL) dwell times are associated with increased risk of central line associated blood stream infections (CLABSIs). Removal of invasive devices when they are no longer clinically indicated is key to CLABSI prevention.

Table 1

**Figure:**
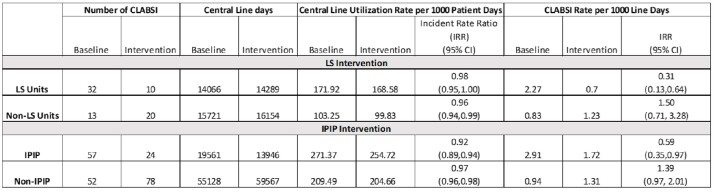

Comparison of the Device Review Programs Impact on Central Line Utilization Rates and CLABSI Rates

**Methods:**

Our health system designed two quality improvement initiatives intended to serve as external review and feedback processes on the necessity of invasive devices. Line Stewardship (LS) was initiated March 2022 in Hospital A and later implemented in 6 acute care facilities. A team consisting of infection preventionists (IP), vascular access nurses and nurse leaders performed chart reviews to determine if CLs met institutional criteria for necessity. If no indication for the device was found, the clinical team was contacted to recommend removal. The second program was called Infection Prevention Integration Program (IPIP), which had a stepwise rollout throughout 2022 in 5 acute care facilities. This program involved IPs joining multi-disciplinary rounds to make recommendations on device removals.

**Results:**

Hospital A piloted the LS program in 8 units and during the first year, removed 398 CLs, and saw a reduction in the CLABSI rate compared to the year prior (Table 1). Similarly, implementation of IPIP led to a reduction in the CLABSI rate. Both non-LS and non-IPIP units reported an increase in CLABSI rates during the intervention period compared to baseline (Table 1).

**Conclusion:**

Both programs saw improvements in CLABSI rates, achieving the goal of reducing infections. IPIP units saw a larger decrease in the utilization rate compared to LS units. This may be due to differences in the criteria used to determine eligibility for line review. Also, there were differences in unit selection; general wards were selected in LS facilities and intensive care units in IPIP facilities. Both LS and IPIP teams noted benefits of the program beyond line removal in improved attention of staff to overall infection prevention practices. Given the increases seen on non-LS and non-IPIP units, it is important to ensure resources aren’t being diverted away from non-intervention units. While both interventions are time and resource intensive, they led to a reduction in CLABSI burden justifying the necessary institutional support.

**Disclosures:**

**All Authors**: No reported disclosures

